# Perceptions and factors affecting pharmaceutical market access: results from a literature review and survey of stakeholders in different settings

**DOI:** 10.3402/jmahp.v4.31660

**Published:** 2016-09-27

**Authors:** Semukaya Sendyona, Isaac Odeyemi, Khaled Maman

**Affiliations:** 1 Creativ-Ceutical Ltd, London, UK; 2 Astellas Pharma Europe Ltd, Surrey, UK

**Keywords:** definition, market access, factors, successful, drug development, review, survey

## Abstract

**Background:**

A change in the pharmaceutical environment has occurred from previously only needing to convince regulators of a product's safety and efficacy to obtain marketing authorisation to now needing to satisfy the value perceptions of other stakeholders, including payers, to attain market access for products. There is thus the need to understand the concept of market access that may be defined as ‘the process that ensures the development and commercial availability of pharmaceutical products with appropriate value propositions, leading to their prescribing and to successful uptake decisions by payers and patients, with the ultimate goal of achieving profitability and best patient outcomes’. The aim of this research therefore was to explore the understanding of market access among various stakeholders and how their understanding of this concept could improve patient access to pharmaceutical products.

**Methods:**

A literature review was conducted on MEDLINE by using the term ‘market access’ to find articles with explicit definitions of market access for pharmaceutical products; non-peer–reviewed and other grey literature sources were also examined. A paper-based interview survey was also conducted in three different settings. The respondents were asked about what factors they think contribute to the successful development of pharmaceutical products, as well as their definition of market access for these medicines.

**Results:**

The peer-reviewed literature review did not reveal appropriate comprehensive definitions for market access, although several definitions were proposed from the non-peer–reviewed literature. These definitions ranged from basic to detailed. The survey of 110 respondents revealed differing levels of understanding of market access. Factors considered to influence successful market access, as described by the respondents, included unmet need/burden of disease (68.2%), clinical efficacy (47.3%), comparator choice (36.4%), safety profile (36.4%), and price (35.5%).

**Conclusion:**

The concept of market access is still poorly understood, and the definition varies depending on the stakeholders’ perspectives. For cost-effective products to be developed and made accessible to patients, there is a need for wider understanding of market access and the value perspectives of the various stakeholders. There is also a need to determine whether and how involved payers should be in the development of pharmaceutical products.

It is widely accepted that activities during research and development, sales, and marketing have been the main drivers of commercial success for pharmaceutical companies. These activities typically involved submitting data on efficacy, safety, and tolerability to the regulatory agencies; and once approved, marketing the drug to the appropriate physicians and pharmacists. Access to the market therefore involved a small set of stakeholders (1). In addition, differences in regulatory requirements between countries make market access difficult (2), and there have been changes in the way healthcare services are provided: 1) patients are increasingly being informed about their conditions and possible treatments, and their perspectives are required or taken into account during the early phase of product development; 2) patient associations have become more active and are able to lobby for public funding of, often costly, curative treatments; and 3) doctors are obliged to follow national prescribing guidelines and may have less freedom to choose treatments (2). Considering the changing pharmaceutical environment, it is necessary to examine the understanding of market access.

Previous reports suggested that one of the factors contributing to successful product development was government support for academic research and development (3). The collaboration between research chemists and the National Institutes of Health in the United States revolutionised pharmaceutical development, making it much cheaper and easier to identify potential drug candidates (3). Other ways of growing the return on investment are by increasing prescription prices, raising awareness through advertising campaigns, and successfully lobbying for patent extensions (4).

Recently, research seems to suggest that the ‘one size fits all’ method of drug development, as used to create blockbusters, is on the decline. Instead, personalised medicine is the new way forward; hence, the need for a broader definition of market access (5). The aim of this personalised approach is to design appropriate treatment for each person's unique needs, taking into account clinical, biological, genetic, environmental, and socioeconomic factors and life styles. This information ideally should allow accurate predictions to be reached about a person's susceptibility to develop disease and response to treatment, and lead to elimination of therapeutic failure and toxicity (5). There is a need for this shift in approach because in many cases, the treatment is not effective, especially in cancer treatments, where 75% of therapy protocols are ineffective (5). This ineffectiveness as well as efficacy compared with standard of care can act as barriers to successful pricing and reimbursement of treatments. Personalised medicine is an opportunity for stakeholders to reap the maximum benefit from drugs by delivering the best treatment (for patients) at a suitable cost (for payers) and reimbursement (for manufacturers).

A working definition of market access is that it is *the process that ensures the development and commercial availability of pharmaceutical products with appropriate value propositions, leading to their prescribing and to successful uptake decisions by payers and patients with the ultimate goal of achieving profitability and best patient outcomes* (6). This multi-perspective definition has been described as encompassing three dimensions and 10 variables (see Table 1).

**Table 1:** Multi-perspective definition of market access showing dimension and variables^a^

Stakeholders	Manufacturers Regulators Physicians Payers Patients
Outcomes	Manufacturers Patients
Life-cycle position	Pre-launch Peri-launch Post-launch

a From Odeyemi (6).

With this understanding, the aims of this research were to 1) develop a robust definition for market access and 2) determine how market access could influence what factors lead to the successful development of pharmaceutical products.

## Methods

### Literature review

A search of the peer-reviewed literature was conducted on MEDLINE by using the term ‘market access’. Articles had to include an explicit definition of pharmaceutical market access. The search criteria included English language, humans, and publication dates between 1 January 2000 and 1 January 2015. This time frame was chosen to coincide with commencement of the widespread use of health technology assessments. Titles and abstracts were initially screened for relevant articles, and then the full text was reviewed for historical and contemporary definitions of market access. Internet searches, using the Google search engine, of grey literature were also conducted to identify non-peer–reviewed definitions of market access. Furthermore, a specific search of the *Journal of Market Access and Health Policy* was conducted. The references sections of all the articles identified for inclusion were also searched for additional references.

### Survey

A paper-based, qualitative, and structured questionnaire (see Supplementary File 1) was developed to be completed by relevant stakeholders such as healthcare professionals, academics, pharmaceutical industry experts, policy makers, payers, health technology assessors, and consultants in three different settings: an international pharmacoeconomics conference, a market access educational course, and a pharmaceutical company. The questions were developed in collaboration with a market access expert. The aim of the survey was not only to gauge the respondents’ understanding of market access but also their perspectives on factors affecting the successful development of pharmaceutical products, and patients’ and payers’ influences during the product development cycle. Their responses to questions about the patients’ and payers’ influences during the product development cycle are not presented in this article; they will be reported in a follow-up publication.

At a health industry conference setting (ISPOR 2014), two researchers randomly approached attendees, briefly introduced themselves, outlined the purpose of the survey, and then asked them if they would be willing to anonymously respond to the questionnaire. Some of the attendees were approached based on their status as well-known, highly respected key opinion leaders and for their influence in their respective fields. The researchers then asked the questions, recording the responses on the question sheet. In the educational setting, the respondents completed the questionnaire on their own after attending a session on market access; in the pharmaceutical setting, respondents were approached after internal meetings and asked to complete the survey.

The responses to the question *List five factors that you believe would influence the development of a successful pharmaceutical product* elicited 17 response categories (see Supplementary File 2). The responses to the question *What is your definition of market access regarding pharmaceutical products?* were coded into the three dimensions – stakeholders, outcomes, position in life-cycle – and 10 variables in line with the definition proposed by Odeyemi (6) (see Supplementary File 3).

It is important to note that the variables and dimensions were not mutually exclusive. A respondent could, for example, name patients and payers in their definition and both would be counted in their response.

All questionnaires were completed anonymously, and none of the respondents received payment. Each interview was anticipated to last no longer than 10 min. The answers to the questionnaire were analysed using descriptive statistics. Because all the questions were open ended, the responses were first recorded verbatim and then coded by the researchers after discussions with a market access expert to aid input and analysis in Excel (Microsoft).

## Results

### Literature review

In total, 110 articles were identified from the literature review: 55 of these articles were excluded based on not meeting the inclusion criteria and 22 of these articles were excluded based on a full-text review. In addition, 10 articles were obtained from the Google search and two from *Journal of Market Access and Health Policy*, yielding 45 articles in total for analysis.

After a full-text review, only four of the articles included a definition of market access (1, 6–8) (Table 2). Most of the remaining 41 articles described the evolution of market access, from solely focusing on obtaining regulatory approval, to the current situation that requires demonstration of a product's quality, safety, and efficacy, but often comparative clinical- and cost-effectiveness data and the reimbursement agreement by a payer.

**Table 2:** Definitions of market access identified in literature review

Source	Definition of market access
Harvard Center for International Development; Odeyemi (6)	*… an umbrella term for a number of measures that a country may use to restrict imports*.
Health Access Strategies; Odeyemi (6)	*… a company's ability to secure funding in alignment with the commercial strategy*.
pharmaLevers GmbH; Odeyemi (6)	*… a stakeholder tailored, multidisciplinary, aligned approach to accelerate market uptake by optimising value demonstration, pricing and reimbursement*.
Hu; Odeyemi (6)	*… usually refers to the processes companies take to get their drugs commercially available within a given community, country, or global region*.
Odeyemi (6)	*… the process whose function is to ensure the development, commercial availability and successful uptake of pharmaceutical products*.
Robinson (7)	*… strategic planning to ensure that the new products are adopted by key stakeholders and therefore accessible upon approval and launch with minimum barriers to use*.
Wight (8)	*… the process to ensure that all appropriate patients who would benefit, get rapid and maintained access to the brand, at the right price*.
Kumar et al. (1)	*… a process that ensures all appropriate patients have rapid and continued access to the product at the right price*.
	*Market access involves engaging with all components of a market and with different stakeholders who impact the overall product commercialisation process*.
	*Market access involves various processes and activities for engaging with a diverse set of stakeholders*.

### Survey

In total, 110 respondents answered the questionnaire: 48 at the conference, 45 at the educational course, and 17 at the pharmaceutical company. Of the respondents, 76 (69%) were pharmaceutical industry experts, 13 (12%) were pharmaceutical consultants, 11 (10%) were academics, 8 (7%) were policy makers or health technology assessors, and 2 (2%) were healthcare professionals.

Q2. List five factors that you believe would influence the development of a successful pharmaceutical product?

Across all professions, the top five factors believed to influence development of a successful pharmaceutical product were as follows: unmet need/burden of disease, clinical efficacy, comparators, safety, and price (Table 3). The responses according to profession are detailed in Table 3.

**Table 3:** Responses to question on factors that influence development of a successful pharmaceutical product by profession

Profession	Factor
All	• Unmet need/burden of disease (68.2%)
	• Clinical efficacy (47.3%)
	• Comparators (36.4%)
	• Safety (36.4%)
	• Price (35.5%)
Pharmaceutical industry	• Unmet need/burden of disease (71.1%)
	• Clinical efficacy (48.7%)
	• Comparators (40.8%)
	• Safety (35.5%)
	• Price (34.2%)
Academic	• Unmet need/burden of disease, clinical efficacy (45.5%)
	• Safety, price, clinical effectiveness, cost-effectiveness (36.4%)
	• Comparators, research and development, good trial design, patient view/quality of life (18.2%)
Healthcare professional	• Comparators, price, clinical effectiveness, cost-effectiveness, payer/policy maker's perception, innovation (50%)
Policy maker/health technology assessor	• Unmet need/burden of disease (75%)
	• Early dialogue (62.5%)
	• Clinical efficacy, Price (50%)
	• Safety, research and development (37.5%)
Consultant	• Unmet need/burden of disease (76.9%)
	• Clinical efficacy, safety (46.2%)
	• Comparators (38.5%)
	• Price, research and development (30.8%)

Q7. What is your definition of market access regarding pharmaceutical products?

Respondents offered a wide range of definitions of market access regarding pharmaceutical products (Table 4), each with unique insight. Some of the responses were vague, whereas others were detailed. One respondent summarised the difficulty in defining market access by saying that, *It would be so difficult to define ‘market access’ in pharmaceutical products: considering the implication products may have on the wider healthcare market, understanding the impact the changing healthcare market will have on the product, and preparing a positive healthcare environment which suggests uptake of the product*. Examples of appropriate market access definitions can also be found in Table 4.

**Table 4:** Selected responses to question on definition of market access

Nature	Definition
Vague	*It's a long process from the idea to the launch and post marketing*.
Vague	*How to get payers to pay for an expensive drug*.
Vague	*Process by which pharmaceutical companies get drugs to the market, so it becomes available for the patients*.
Vague	*Market access is how you influence all stages of a product cycle to ensure the largest number of patients get access to your product over the full life-cycle of a product*.
Concise	*For pharmaceutical companies: obtaining and maintaining access to drug[s] in [the] planned population and at [the] optimal price with minimal budget impact*.
Concise	*Process ensuring all appropriate patients who would benefit from a drug get rapid and maintained access to the drug at an affordable price with minimal barriers to use*.
Concise	*Balancing the needs and interests of three (major) players: economic interests of pharmaceutical companies vs. patient interests in best care vs. budgets and legal constraints of health system*.

#### Respondents’ perspectives on market access dimensions

Odeyemi (6) proposed three dimensions – stakeholders, outcomes, and life-cycle position – and 10 variables – patients, payers, pharmaceuticals, physicians, regulators, pharmaceutical, patient, pre-launch, peri-launch, post-launch – as fundamental elements to an appropriate definition of market access.

**Fig. 1:**
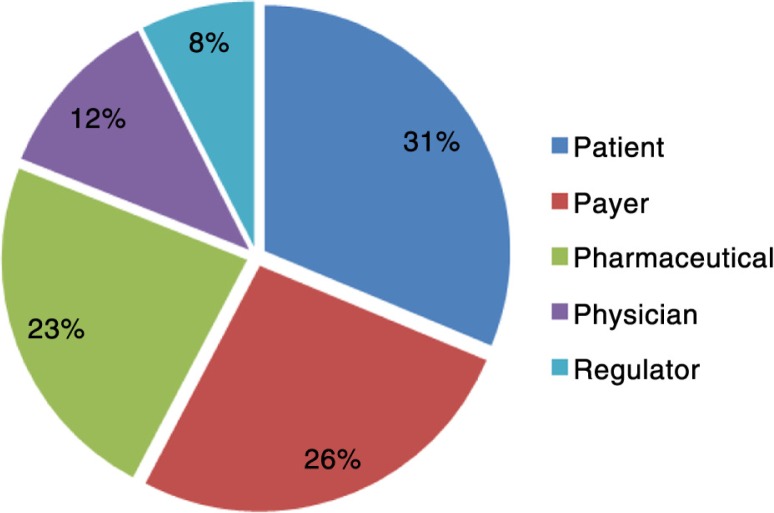
Percentage of respondents including stakeholders in their market access definition.

*Stakeholders*. The percentage of respondents who included the stakeholders in their definition of market access is shown in Fig. 1. As the end user of treatments, it makes sense that most of the respondents named patients as integral to market access. There were different ways that respondents perceived patients as the most important stakeholder. Responses included *Product availability, affordability, and acceptability (packaging/posology/patient friendly) for patients as primary end users*’. and *Make treatments available for those who really need them for as long as they need them or benefit from them despite the patient ability to pay for it and make patients aware of those treatments’ availability in order to seek them/use them*.

With payers seeing their influence increase, especially being depicted as the gatekeepers to drug access, respondents named them as the next most important stakeholders. Their role in market access was mentioned in different ways, such as *The capability of an organisation to understand and anticipate payer needs and deliver value to this customer group*, and also as *Gaining access on the market. A product will have good market access if it: has the best recommendations, is included on formularies, has no price barriers*.

Several respondents also recognised the importance of pharmaceutical companies in their definition, with one respondent defining market access as *Optimis[ing] access of a drug along the development life-cycle with the appropriate target product profile (adopt target product profile along development life-cycle), developing early access propositions with health economic evaluation*.

Physicians are often found at the penultimate stage to drug use by patients, and market access was loosely described as *[A] connection of three parties: patient, industry, physician. and Patients are diagnosed/treated in fastest time by [the] appropriate drug/treatment*. Most of the respondents indirectly mentioned physicians by saying *drugs being prescribed* possibly with the belief that only physicians can prescribe drugs, so negated to specifically mention them.

The role of regulators in market access was appreciated by the lowest number of respondents. Perhaps this low number is because some still see their role just as providing regulatory approval or marketing authorisation that later leads to market access. However, Eichler et al. suggest that in the future, a two-stage process between the licensing and reimbursing decisions will occur (9). First, an assessment of relative efficacy or comparative effectiveness research will be made, with a focus exclusively on the medical consequences of competing treatment options. This assessment would then be followed by a second analysis of context-specific economic consequences undertaken by individual payers (9). One respondent defined market access as *Varies. [It] should be everything that is done to get the product to be prescribed (regulatory etc.)*, whereas another said *The discipline governing all matters that can eventually impact a products’ access to a market. In the pharmaceutical sector, this notably entails regulatory/licensing matters, rules governing the setting of a product's price and possibly funding/reimbursement*.

#### Outcomes

**Fig. 2:**
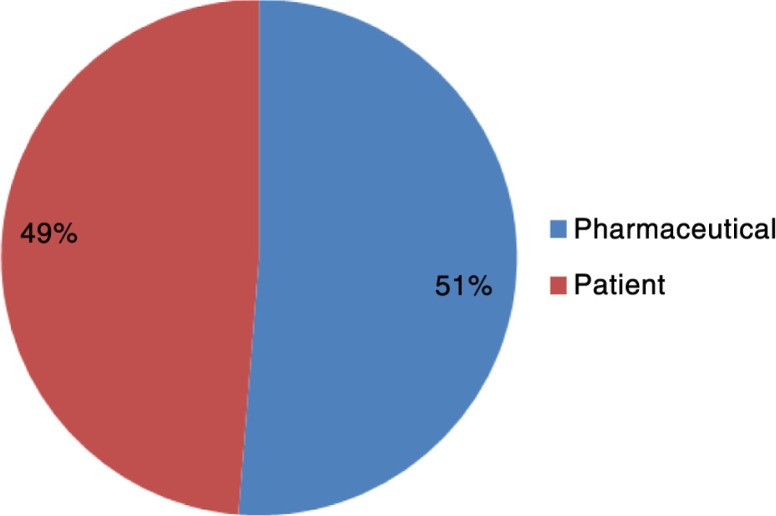
Percentage of respondents including outcomes in their market access definition.

How respondents perceived the importance of pharmaceutical and patient outcomes when defining market access is shown in Fig. 2. Pharmaceutical industry outcomes generally include making profits by achieving a high number of sales, obtaining a premium price, securing reimbursement, or a combination of the three for the manufacturer. Market access was simply described as *Obtain[ing] the commercialisation of a product* and *[The] process where manufacturers seek reimbursement to launch a product onto the market*.

Patient outcomes mostly include the improvement of health-related quality of life, for example, the improvement of health outcomes such as the reduction in severity of symptoms or an improvement in the method of administration of the treatment. Market access was defined as *Define high value product to patient[s] in need and improve their health* and *[Market Access] can be defined as the uptake of the product by patients that need it, and paid by the insurance/social security, providing efficacy/effectiveness where other products are unable to*.

#### Life-cycle position

**Fig. 3:**
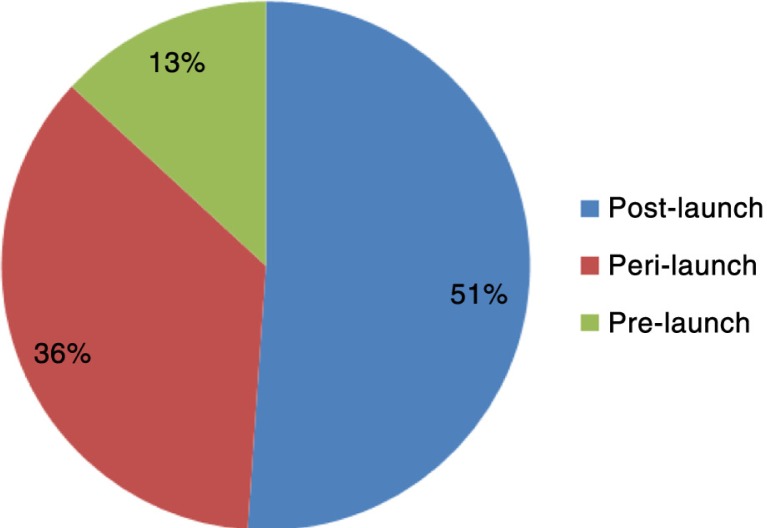
Percentage of respondents including life-cycle position in their market access definition.

The life-cycle position in which respondents deemed market access activities to most take place are shown in Fig. 3. Odeyemi (10) proposes three activities take place during the pre-launch phase: target product profile, product development plan, and clinical trial design. Respondents’ answers varied from *Try[ing] to have knowledge of [the] economic environment, and regulations around drugs* to *All the phases that lead to effective marketing of a product*. More detail was found in another definition, *[Market access is a way to] optimise access of a drug along the development life-cycle with the appropriate target product profile (adopt target product profile along development life-cycle), [and] develop[ing] early access propositions with health economic evaluation*.

Odeyemi (10) also advocates three activities take place during the peri-launch phase: regulatory approval, pricing, and launch plan. Definitions included *[A] process of launching and commercialisation, getting approval and reimbursement* and *Understand[ing] the value of drugs and communicat[ing] this to payers and patients, not only in terms of efficacy, but also budget impact and cost effectiveness, after market authorisation*.

Odeyemi (10) finally suggests three activities take place during the post-launch phase: positioning, detailing, and reimbursement/formulary submission. Predictably, most respondents said market access was a post-launch activity. Market access was defined as *[A] process where manufacturers seek reimbursement to launch a product onto the market* and *Does not stop with launch of product. [It] depends on successful uptake of drug afterwards, positive recommendation, reimbursement actually available on market*. Although not specifically mentioning post-launch, it is indirectly suggested in the responses.

### Respondents’ perspectives on market access variables

In general, only 2 (2%) of 110 respondents alluded to all 10 variables in their definition: a respondent from academia vaguely defined market access is *All aspects of [the] process to secure use and reimbursement of a new product*, and a more thorough definition from a pharmaceutical industry expert as *All actions that will help a pharmaceutical product to be accessible on the market. The market concerns all stakeholders who have an influence inside and outside of the sponsor of the drugs. On one side: regulatory, clinical development, medical, commercial, health economics and outcomes research, market access, [while] on the other: payers, health authorities, patient associations, etc*.

Ten respondents (9%) failed to mention any variables in their response, such as *The way to enter the market smoothly and to be accepted by the market*. Two respondents did not answer the question at all. Most respondents gave between four and six variables: 17 (16%) gave four such as *Analysis of structures and organisation/processes and implementation of services that make the medicine available to patients*. and *It may be the process to ensure that all the patients who are likely to respond to the drug can get the drug with the appropriate price*; 15 (14%) gave five variables, for example, *Process to detect the gaps or the factors which determine the pharmaceutical products use. Developing a strategy and giving a solution to patients for having better access to drugs* and *Collaboration among payer, pharmaceutical companies, and physicians to make products available in a good environment to patients* and 17 (16%) gave six variables, including *Getting the right drug to the right patient at the right price* and *A drug that is approved needs to be made available to the patients who need it. Market access is dealing with all activities required to reduce barriers to access (i.e., affordable price, reimbursement, straightforward administration)*.

None of the respondents gave nine variables, although two (2%) did give eight variables: *Understand the value of drugs and communicate this to payers and patients, not only in terms of efficacy, but also budget impact and cost effectiveness, after market authorisation*. and *Moment of discussion between stakeholders (patient association groups, pharma, regulators) aiming to find real value of a product, making sensible uptake decisions*.

### Market access understanding by respondents’ professional background

Academics mostly defined market access as involving payer, pharmaceutical industry, and patient stakeholders, almost equally taking into account pharmaceutical industry and patient outcomes (64% and 55%), and taking place mostly during the post-launch (91%) and peri-launch (73%) phases (Table 5).

**Table 5:** Frequency (%) of variables named in academics’ definition of market access

Dimension	Variable	Frequency (%)
Stakeholders	Payer	8 (72.73)
	Pharmaceutical	7 (63.64)
	Patient	6 (54.55)
	Regulator	3 (27.27)
	Physician	2 (18.18)
Outcomes	Pharmaceutical	7 (63.64)
	Patient	6 (54.55)
Life-cycle position	Post-launch	10 (90.91)
	Peri-launch	8 (72.73)
	Pre-launch	5 (45.45)

Pharmaceutical consultants also defined market access as involving pharmaceutical industry (77%), payer (69%), and patient stakeholders (54%), equally incorporating pharmaceutical industry and patient outcomes (both 38%), and mostly occurring in the post-launch (77%) phase (Table 6).

**Table 6:** Frequency (%) of variables named in pharmaceutical consultants’ definition of market access

Dimension	Variable	Frequency (%)
Stakeholders	Pharmaceutical	10 (76.92)
	Payer	9 (69.23)
	Patient	7 (53.85)
	Physician	3 (23.08)
	Regulator	3 (23.08)
Outcomes	Pharmaceutical	5 (38.46)
	Patient	5 (38.46)
Life-cycle position	Post-launch	10 (76.92)
	Peri-launch	6 (46.15)
	Pre-launch	2 (15.38)

Although being too few to draw comparable conclusions, healthcare professionals defined market access as including all stakeholders, with a focus on pharmaceutical outcomes, and with activities around market access occurring during the post-launch phase (Table 7). Pharmaceutical industry experts identified patients as the key stakeholders in market access (68%), taking into account both patient (33%) and pharmaceutical (30%) outcomes, with market access activities mostly occurring in the post-launch (59%) phase (Table 8).

**Table 7:** Frequency (%) of variables named in healthcare professionals’ definition of market access

Dimension	Variable	Frequency (%)
Stakeholders	Pharmaceutical	1 (50.00)
	Regulator	1 (50.00)
	Payer	1 (50.00)
	Physician	1 (50.00)
	Patient	1 (50.00)
Outcomes	Pharmaceutical	1 (50.00)
	Patient	0
Life-cycle position	Post-launch	2 (100.00)
	Pre-launch	0
	Peri-launch	0

**Table 8:** Frequency (%) of variables named in pharmaceutical industry experts’ definition of market access

Dimension	Variable	Frequency (%)
Stakeholders	Patient	52 (68.42)
	Payer	36 (47.37)
	Pharmaceutical	31 (40.79)
	Physician	18 (23.68)
	Regulator	9 (11.84)
Outcomes	Patient	25 (32.89)
	Pharmaceutical	23 (30.26)
Life-cycle position	Post-launch	45 (59.21)
	Peri-launch	34 (44.74)
	Pre-launch	10 (13.16)

Policy maker/health technology assessors mostly defined market access as incorporating payers (75%), patients (63%), and pharmaceutical industry (50%) stakeholders, with twice as many respondents saying pharmaceutical outcomes than patient outcomes (50% vs. 25%) were important, and happening during the post-launch (38%) phase (Table 9).

**Table 9:** Frequency (%) of variables named in policy maker/health technology assessors’ definition of market access

Dimension	Variable	Frequency (%)
Stakeholders	Payer	6 (75.00)
	Patient	5 (62.50)
	Pharmaceutical	4 (50.00)
	Physician	2 (25.00)
	Regulator	1 (12.50)
Outcomes	Pharmaceutical	4 (50.00)
	Patient	2 (25.00)
Life-cycle position	Post-launch	3 (37.50)
	Pre-launch	1 (12.50)
	Peri-launch	1 (12.50)

## Discussion

### Literature review

To achieve regulatory approval, pharmaceutical companies need to demonstrate that a product is safe, effective, and meets quality standards. However, to ensure successful market access, they also need to demonstrate the product's value during pricing and reimbursement negotiations.

From the literature review, market access was loosely described as the process to ensure that all appropriate patients who would benefit get rapid and maintained access to the drug, at an affordable price. Although there were a handful of meaningful definitions, none completely took into account all the various stakeholders, outcomes, and phases in the product life-cycle.

Market access is not only about satisfying payers as stakeholders (through negotiating price discounts) but also about satisfying patients (through the appropriate selection of the most suitable patients for treatment) (11). The clear driver for successful market access is the generation of a suitably robust evidence base for good decision making, such that healthcare resources are used for the maximum benefit of patients (12). The overarching market access strategy for a novel therapy therefore requires demonstration of benefit and value in clinical studies and also in routine clinical practice (13). A common theme in the Kumar et al. description of market access is the presence of stakeholders and the need to satisfy their differing needs to initially gain, and then maintain market access (1).

### Survey

Unmet medical need/burden of disease was considered to be the most important factor influencing the development of a successful product: the higher the need for a treatment in a certain disease area, the more likely it should be successful. Surprisingly, research and development was not deemed as important, despite the obvious need for it to inform manufacturers’ decision makers about the development of the new treatment. Early dialogue ought to have been higher up the list too, because it would enable manufacturers to focus more on the aspect of the new treatment that would be of interest to the differing stakeholders. It was unusual that good trial design was rated low compared to clinical efficacy, comparators, and safety because these factors are all joint considerations.

Because the majority of respondents were pharmaceutical industry experts, the level of congruence with the total population is expected. For academics, the expectation was that research and development would be the most frequently named factor; although this outcome was not the case, it was among the top five.

Not surprisingly, 63% of policy makers are interested in early dialogue, because it would be beneficial to all stakeholders involved in health technology assessments. What is interesting is that twice as many respondents said price was important compared with cost effectiveness (50% vs. 25%). One would have thought that these two factors would be closely linked. Perhaps the respondents imagine that outright price has more of an effect on decision makers than cost effectiveness. This could be due to price generally being fixed as an input in economic evaluations (and therefore not flexible to change), whereas other factors can alter the cost effectiveness of a medicine (e.g., the population, line of treatment, duration of treatment).

The survey revealed that market access involves patients and payers as stakeholders, with a view of satisfying both patient outcomes (e.g., improvement in quality of life) and pharmaceutical outcomes (e.g., increasing profits) after the pharmaceutical product has been launched.

In terms of the stakeholders involved in market access, regulators being seen as the least important stakeholders would, in the past, not have been surprising. But currently, regulators and payers are perceived as having overlapping influence on market access, so it would have been expected that respondents named these two more equally. Although it is understood that patients are the most important stakeholder, surprisingly their outcomes (quality of life, patient satisfaction) are almost as important as those of the pharmaceutical industry (profits). At minimum, the difference in outcome importance should have mirrored the difference in stakeholder importance. Finally, for the life-cycle position, as with stakeholders, this would not have been a surprise in the past, but considering a change in attitude about what factors lead to the commercial success of a product, it would be expected that successful market access also depends on activities in the pre- and peri-launch phases of the life-cycle.

The common themes in the most appropriate definitions are that communication among all stakeholders is very important and that all should benefit (albeit in different ways), for example, the pharmaceutical industry may attain the right price for treatment, patients may have minimal barriers to accessing the best treatments, and payers may be charged an acceptable price for the treatments. However, the majority of responses neglected to mention when during the product life-cycle the communication should take place. It is not only important to know which stakeholders with which to communicate but also to know when to communicate with them to optimise the product development cycle.

As can be seen in a sample of the responses, there is a wide discrepancy in different respondents’ understanding of market access. Several descriptions were very basic and generic, with no mention of elements specific to the pharmaceutical industry, whereas others were very detailed. The difference in the levels of detail provided by respondents also helps to emphasise that even within the same professional environment, some experts have a better grasp of market access than others.

A common understanding of what is meant by market access is essential to the development and accessibility of medicines and devices that will benefit patients.

### Limitations

#### Literature review

The literature review focused on articles published starting in 2000; however, none of the reviewed articles referred to any published work before 2000, so it is unlikely that any relevant information regarding a definition was overlooked. A possible limitation could be that only articles published in English were sought; however, a relevant non-English language article could reasonably be expected to be referenced in an English article.

#### Survey

The survey was answered by a small number of respondents, so no statistical inferences could be made about the findings. More participants, especially representing patients and regulators, from wider professional backgrounds would provide more meaningful results. The survey made use of open-ended questions to capture as much detail as possible from respondents. These responses were then coded to aid analysis. It is possible that some of the details in the responses could not be codified and were therefore lost. This type of omission could be lessened if there were validated codes. Also, in the international conference setting, respondents were approached and asked to take part without prior warning, and several people declined to take part. As was seen in some of their responses, it is possible that respondents did not have enough time to thoroughly consider their answers to the questions, for example, the importance of good trial design was rated low compared to clinical efficacy, comparators, and safety, which all contribute to good trial design. If the survey were administered to more prepared respondents, it is possible that more people would have responded and more insight could have been obtained.

## Conclusions

The concept of market access is still poorly understood, and the definition varies depending on the stakeholders’ perspectives. The survey described here revealed that a good awareness of unmet need/burden of disease, clinical efficacy, choice of comparators, safety, and price were the most important factors influencing the development of a successful pharmaceutical product. For cost-effective products to be developed and made accessible to patients, there is a need for wider understanding of market access and the value perspectives of the various stakeholders.

As proposed, a multi-perspective definition of market access is *the process that ensures the development and commercial availability of pharmaceutical products with appropriate value propositions, leading to their prescribing and to successful uptake decisions by payers and patients, with the ultimate goal of achieving profitability and best patient outcomes*.

Thus, research is underway to determine whether and how involved payers and other stakeholders should be in the development of pharmaceutical products. This new research should not only define the stakeholders but also elucidate how they should be involved in the different stages of product development.

## Supplementary Material

Perceptions and factors affecting pharmaceutical market access: results from a literature review and survey of stakeholders in different settings

## References

[1] Kumar A, Juluru K, Thimmaraju P, Reddy J, Patil A (2014). Pharmaceutical market access in emerging markets: Concepts, components, and future. J Mark Access Health Policy.

[2] Eichler HG, Pignatti F, Flamion B, Leufkens H, Breckenridge A (2008). Balancing early market access to new drugs with the need for benefit/risk data: A mounting dilemma. Nat Rev Drug Discov.

[3] Thompson RB (2001). Foundations for blockbuster drugs in federally sponsored research. FASEB J.

[4] Gottlieb S (2002). Drug companies maintain ‘astounding’ profits. BMJ.

[5] Qattan M, Demonacos C, Krstic-Demonacos M (2012). Roadmap to personalized medicine. Croat Med J.

[6] Odeyemi I (2014). Demystifying pharmaceutical market access.

[7] Robinson S (2010). Market access-the definition depends on the viewpoint. Evidence Matters.

[8] Wight C (2012). The true meaning of market access? Understanding fully the words that define market access is the first step on the route to success.

[9] Eichler HG, Bloechl-Daum B, Abadie E, Barnett D, Konig F, Pearson S (2010). Relative efficacy of drugs: An emerging issue between regulatory agencies and third-party payers. Nat Rev Drug Discov.

[10] Odeyemi I (2014). Developing a pharmaceutical market access strategy: The KIPs framework.

[11] Jonsson B, Wilking N (2012). Cancer vaccines and immunotherapeutics: Challenges for pricing, reimbursement and market access. Hum Vaccin Immunother.

[12] Payne K, Annemans L (2013). Reflections on market access for personalized medicine: Recommendations for Europe. Value Health.

[13] Rofail D, Taylor F, Regnault A, Filonenko A (2011). Treatment satisfaction instruments for different purposes during a product’s lifecycle: Keeping the end in mind. Patient.

