# Supplementary material for: Perceptions and factors affecting pharmaceutical market access: results from a literature review and survey of stakeholders in different settings
Source: J Mark Access Health Policy. 2016 Sep 27;4:10.3402/jmahp.v4.31660. doi: 10.3402/jmahp.v4.31660 (PMC5040822; doi:10.3402/jmahp.v4.31660)
Supplement: Perceptions and factors affecting pharmaceutical market access: results from a literature review and survey of stakeholders in different settings [file JMAHP-4-31660-s001.docx]

# SUPPLEMENTARY MATERIAL

**1. Survey of payers’ and patients’ perspective into pharmaceutical market access in the three different settings.**

1. What is your current professional background?
   1. Pharmaceutical industry
   2. Academic
   3. Healthcare professional
   4. Policy maker
   5. Payer (e.g. health insurance/national health service)
   6. Health Technology Assessment body
   7. Consultant
2. List 5 factors that you believe would influence the development of a successful pharmaceutical product.
3. Do payers currently have a role in pharmaceutical development? If so, what?
4. Do patients currently have a role in pharmaceutical development? If so, what?
5. How should the payer’s perspective influence the development of a pharmaceutical product?
6. Early phase
7. Post marketing
8. How should the patient’s perspective influence the development of a pharmaceutical product?
9. Early phase
10. Post marketing
11. What is your definition of market access regarding pharmaceutical products?

**2. Categorisation and explanation of interviewees’ responses.**

- Unmet need/Burden of Disease
  - Whether the proposed treatment targets a condition that has a high burden of disease or unmet need in society
- Clinical efficacy
  - How well the drug performs during clinical trials
- Comparators
  - Whether clinical trial comparators were appropriate
- Safety
  - Whether the drug has an acceptable safety profile
- Price
  - How much the drug will cost to payers/physicians/health insurance providers
- Research and development
  - What work is done leading up to the selection of a viable drug candidate
- Clinical effectiveness
  - How well the drug performs in real world use
- Cost effectiveness
  - How well the drug is an appropriate use of health care resources (e.g. money)
- Payer/policy makers perception
  - How payers/policy makers are likely to view the drug (e.g. its suitability for the population)
- Innovation
  - The level of innovation behind the new drug (e.g. first-in-class, me-too, generic)
- Market Access
  - How well the new drug is marketed and promoted to differing stakeholders
- Good trial design
  - How well the clinical trials are planned and executed to arrive at the data
- Early dialogue
  - Whether there is early communication with other stakeholders during the drug development process
- Patient view/Quality of Life
  - Whether the needs of patients (especially their quality of life) are taken into account during drug development
- Product profile
  - The details of the drug itself (e.g. dosing schedule, posology, method of administration)
- Regulation
  - The laws and regulations in a specific location that may enable or restrict the use of the drug
- Population
- The people most likely to benefit from the drug (which could be a smaller population than that matching the indication)

**3. Dimensions and variables coding in response to question “What is your definition of market access regarding pharmaceutical products?”**

- Stakeholders
  - Pharmaceutical
    - Manufacturers of the treatment
  - Regulator
    - Provide the marketing authorisation for new treatments
  - Payer
    - Any group or body that pays for treatments
  - Physician
    - Medical professionals who prescribe or recommend treatments to patients
  - Patient
    - End users of the treatments
- Outcomes
  - Pharmaceutical
    - Generally sales and profits accrued from the sale and utilisation of their treatments
  - Patient
    - Health benefit realised through the use of the treatment, such as improved quality of life, cure from disease, reduction in disease symptoms
- Position in life-cycle
  - Pre-launch
    - Includes all the steps leading to regulatory approval, from deciding the target product profile, product development plan, and clinical trial design, to execution
  - Peri-launch
    - Includes all the steps leading to product launch, from gaining regulatory approval, and determining price, to agreeing the launch plan
  - Post-launch
    - Includes all the steps after product launch, from the positioning of the product, and detailing, to reimbursement and inclusion on formularies
